# Potential of macronutrients and probiotics to boost immunity in patients with SARS-COV-2: a narrative review

**DOI:** 10.3389/fnut.2023.1161894

**Published:** 2023-05-15

**Authors:** Afrouz Mardi, Aziz Kamran, Farhad Pourfarzi, Maryam Zare, Azadeh Hajipour, Saeid Doaei, Negin Abediasl, Daniel Hackett

**Affiliations:** ^1^Department of Public Health, School of Health, Ardabil University of Medical Science, Ardabil, Iran; ^2^School of Medicine and Allied Medical Sciences, Ardabil University of Medical Sciences, Ardabil, Iran; ^3^Digestive Disease Research Center, Ardabil University of Medical Sciences, Ardabil, Iran; ^4^Department of Nutrition, Khalkhal University of Medical Sciences, Khalkhal, Iran; ^5^School of Health, Qazvin University of Medical Sciences, Qazvin, Iran; ^6^Department of Community Nutrition, Faculty of Nutrition and Food Technology, National Nutrition and Food Technology Research Institute, Shahid Beheshti University of Medical Sciences, Tehran, Iran; ^7^Physical Activity, Lifestyle, Ageing and Wellbeing Faculty Research Group, School of Health Sciences, Faculty of Medicine and Health, The University of Sydney, Lidcombe, NSW, Australia

**Keywords:** protein, lipid, carbohydrate, probiotic, immune system, SARS-CoV-2

## Abstract

Severe Acute Respiratory Syndrome Coronavirus-2 (SARS-COV-2) may cause inflammation and increased cytokine secretion. Dietary factors may play an important role in enhancing the immune responses against infectious diseases such as SARS-COV-2. This narrative review aims to determine the effectiveness of macronutrients and probiotics to improve immunity in SARS-COV-2 patients. Dietary proteins may boost pulmonary function in SARS-COV-2 patients through inhibitory effects on the Angiotensin-converting enzyme (ACE) and reduce Angiotensin (ANG-II). Moreover, omega-3 fatty acids may improve oxygenation, acidosis, and renal function. Dietary fiber may also produce anti-inflammatory effects by reducing the level of high-sensitivity C-Reactive Protein (hs-CRP), Interleukin (IL-6), and Tumor necrosis factor (TNF-α). In addition, some evidence indicates that probiotics significantly improve oxygen saturation which may enhance survival rate. In conclusion, the consumption of a healthy diet including adequate macronutrients and probiotic intake may decrease inflammation and oxidative stress. Following this dietary practice is likely to strengthen the immune system and have beneficial effects against SARS-COV-2.

## Introduction

Severe Acute Respiratory Syndrome Coronavirus 2 (SARS-COV-2) is a viral infectious disease that in the previous 2 years has caused a worldwide pandemic (1). In December 2019 (Wuhan, China) SARS-COV-2 was first reported and characterized as a pandemic in March 2020 by the World Health Organization (WHO) (2). Millions of people worldwide have been infected with SARS-COV-2, while over six million people have died from this disease (3). The symptoms of COVID-19 vary from asymptomatic to mild upper respiratory tract symptoms and can progress to pneumonia. Patients with severe pneumonia rapidly deteriorate over a short period and require advanced medical support (4–6). SARS-COV-2 colonizes the respiratory tract but can also invade the gastrointestinal tract, neurological system, kidneys, and other organs (7, 8). SARS-COV-2 utilizes the host cell membrane receptor ACE2 to enter the virus into lymphocytes, monocytes, pulmonary alveoli, and esophageal epithelial cells (9). The cytokine storm can be induced by increases in the production of pro-inflammatory cytokines, such as IL-6, IL-1, and TNF-α, in which a hyperactive immune response is experienced. The effects of this response include vessel and lung alveoli damage, and systemic organ failure, such as acute respiratory syndrome (ARDS), which has been associated with high rates of mortality (10).

Immunity to viruses can be influenced by several factors such as genetics (11), vaccination history (12), illness, medications, sex (13), stage of life (e.g., pregnancy, infancy, and old age) (14), and diet (15). Specifically, an adequate intake of some dietary components is essential for developing, supplying, and expressing immune responses (16). A balanced diet of macronutrients (i.e., carbohydrates, protein, and fats) has anti-inflammatory effects and can affect different stages of the immune response. Consequently, an unbalanced diet of macronutrients can affect innate and adaptive immunity, hence making people more susceptible to infections and severe clinical conditions (17).

Recent studies reported that critically ill patients with SARS-COV-2 are at high risk for malnutrition (18). In this context, a recent study emphasized the supportive role of dietary supplementation in SARS-COV-2 patients (19). Therefore, following a well-balanced diet in combination with certain dietary supplements may strengthen and optimise the function of the immune system (20). Particular types of supplements shown to have antiviral activities against common respiratory viruses are probiotics. These dietary supplements can regulate cytokine secretion and affect the immune response (20, 21). At present there is limited evidence on macronutrients and probiotics intake in patients with SARS-COV-2. The aim of this narrative review was to examine the effects of macronutrients and probiotics on the immune system in patients with SARS-COV-2.

## Protein

Proteins are essential to life because they facilitate biochemical reactions and enzyme production. They also act as cellular signals in the form of hormones and cytokines (22). An adequate protein intake is necessary for antibody production in the body. An adult requires 0.8 g of protein per kg of body weight to meet the recommended dietary allowance (RDA) (23). Key amino acids for stimulating the immune system include arginine and glutamine (24). Arginine serves as the vital substrate for synthesizing nitric oxide by macrophages and endows them with pro-inflammatory and microbicidal properties (25). As for glutamine, this amino acid is used as an energy source for the immune cells similar to glucose (26).

### The immunomodulatory role of protein in SARS-COV-2

To defeat the barrier and bind the host cells in the alveoli, SARS-COV-2 utilizes ACE2 (membrane-bound enzyme localized on type II pneumocystis) and cellular serine protease transmembrane protease, serine 2 (TMPRSS2) to prime and replicate in infected organisms (27). ACE2 is expressed in endothelial cells including the heart, kidneys, and intestines (28). When the SARS-COV-2 has entered ACE2-positive cells, there is a down-regulation of the enzyme (29). ACE2 usually binds angiotensin II, a molecule recently certified as a critical player in the conventional renin-angiotensin system (RAS) which promotes inflammation, oxidative stress, and apoptosis (23, 30). The pro-inflammatory angiotensin II is converted into the anti-inflammatory angiotensin 1–7 by binding the enzyme to angiotensin receptors, which exerts a pro-inflammatory effect (23). Therefore, higher production of angiotensin 1–7 can protect the endothelial barriers of the lungs, kidneys, heart, and intestines supporting patients with viral respiratory tract infections from harmful inflammation (31). Dietary peptides with inhibitory effects on the angiotensin-converting-I enzyme (ACE-I) can significantly reduce angiotensin (ANG-II). Dietary-derived peptides can contribute to the downstream ANG-II function. The lactoferrin-released tetra-peptide RPYL (62% of concentration 300 μmol/l) has been found to have inhibitory effects on ANG-II vasoconstriction (32). Additionally, lysine has been found to down-regulate the level of ANG-II (33).

Asymptomatic and mild SARS-COV-2 patients treated with lactoferrin (a milk-derived 80-kDa glycoprotein) had faster clinical recovery compared to untreated patients (34). Therefore, lactoferrin (Lf) may be a safe treatment combined with other therapies for COVID-19 patients. A retrospective study focused on the antiviral activity of Lf found that SARS-CoV-2 RNA was negativized faster in Lf treated versus untreated patients (35).

**Figure 1:**
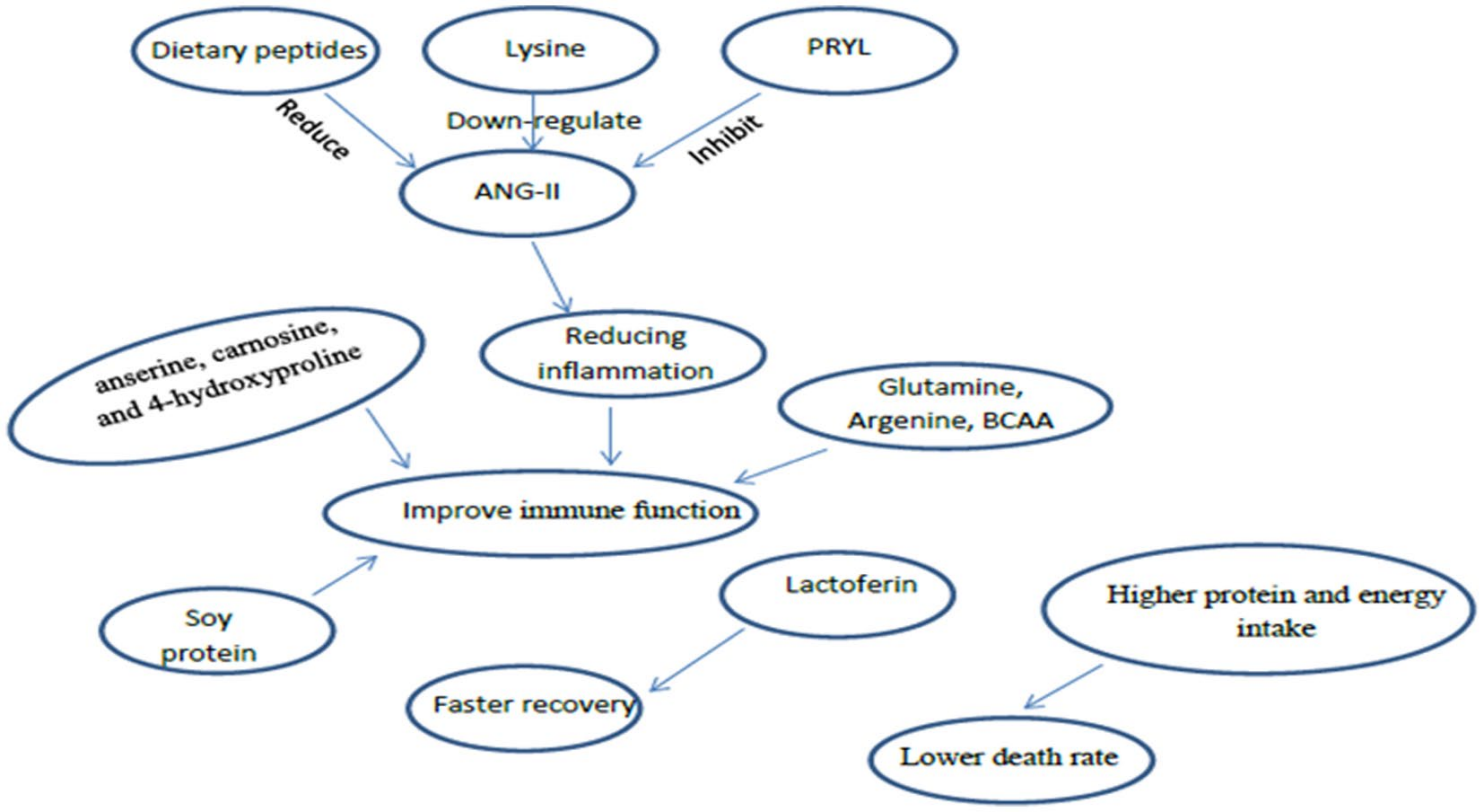
The immunomodulatory role of protein in SARS-COV-2.

Evidence from previous review studies indicates that a diet that positively impacts immune function contains adequate amounts of protein, particularly glutamine, arginine, and branched-chain amino acids (BCAAs) (36, 37). Dietary intake of anserine, carnosine, and 4-hydroxyproline has been shown to improve the body’s immunological defenses against bacteria, fungi, parasites, and viruses (38). Soy protein enhances cellular immunity through a reduction in TNF-α concentrations (39). However, primary sources of amino acids include fish, meat, and poultry. Therefore ensuring that the intake of peptides is optimised may improve respiratory function, symptoms, and fatigue in SARS-COV-2 patients (40). Support for this recommendation is shown by impaired immunity with protein-deficient diets, which would increase the risk of COVID-19 infection (26) (Figure 1).

Patients with SARS-COV-2 receiving enteral nutrition for >7 days where consistent levels of adequate protein and energy are received have a lower death rate compared to patients with suboptimal protein intake (32). Additionally, patients who died of SARS-COV-2 in the hospital were found to have a lower protein and energy intake compared to patients that survived (41). It could be postulated that the intake of protein and energy in critical status patients with SARS-COV-2 hospitalized in ICU are suboptimal. Therefore, it would be prudent that patients with SARS-COV-2 consumed higher protein and energy intake in the early acute phase increasing their chances of survival and lowering the risk of in-hospital mortality.

## Lipid

Dietary intake of fatty acids positively influences the immune response. The two essential fatty acids include omega-3 and omega-6 fatty acids. Additionally, long-chain polyunsaturated fatty acids (PUFA) need to be obtained from foods in the diet since they cannot be synthesized by the human body (42). The omega-3 fatty acids essentially comprise of α-linolenic acid (ALA) from plant sources and docosahexaenoic acid (DHA) and eicosapentaenoic acid (EPA) from fish and seafood sources (43). Anti-inflammatory responses in the body have been shown with the consumption of omega-3 fatty acids (44). In contrast, omega-6 fats mainly provide energy to the body, but the population should ideally receive more omega-3 fats (45). The ratio of omega-6 and omega-3 fatty acids in diets should be between 1:1–4:1.

### The immunomodulatory role of lipids in SARS-COV-2

EPA and arachidonic acid (ARA) compete with the lipoxygenase and cyclooxygenase enzymes to synthesize eicosanoids [prostaglandins (PG), thromboxane (TXA), leukotrienes (LT), hydroxylated fatty acids, and lipoxins] and lipid mediators. ARA is a precursor for pro-inflammatory eicosanoids that are both immunosuppressive and thrombotic (46). EPA converts eicosanoids to antithrombotics. EPA-derived LTB5 has a less chemotactic effect on neutrophils than ARA-derived LTB4. Therefore, EPA has a lower pro-inflammatory response than ARA. LTB4 is a critical pro-inflammatory mediator in cell proliferation and immune response (16).

Omega-3 fatty acids alter the phospholipid bilayer of the cell membrane, thus preventing viral entry. EPA gets incorporated into the plasma membrane and affects the clumping of toll-like receptors. This results in inhibiting signals that activate NF-kB, producing fewer pro-inflammatory mediators, and finally, reducing complications of SARS-COV-2 infection (33, 38). Another mechanism by which Omega-3 fatty acids reduce inflammation levels is *via* the inhibition of leukocyte chemotaxis, expression of adhesion molecules, and interaction between leukocytes and endothelial (26, 40). They also have antiviral effects through the inhibition of influenza virus replication. Furthermore, the use of omega-3 fatty acids is shown to improve oxygenation in SARS-COV-2 patients (47). Consistent with this finding is evidence that omega-3 fatty acids *via* parenteral and enteral means could improve oxygenation in SARS-COV-2 patients, although currently, the evidence is insufficient (48).

Supplementing with omega-3 fatty acids might be beneficial in managing inflammation-mediated clinical symptoms in SARS-COV-2 patients (49). Omega-3 fatty acids are precursors of specialized pro-resolution mediators (SPMs) that present long-lasting analgesic and anti-inflammatory effects (50). As such, omega-3 fatty acids are used for the clinical management of patients with SARS-COV-2. Resolvins, protecting, and maresins of omega-3 fatty acids can inactivate polymorphonuclear leukocytes and stimulate the movement of non-inflammation leukocytes, thereby eliminating programmed cell death (32). Omega-3 supplementation was found to have promising effects on acidosis and renal function, possibly improving clinical outcomes in SARS-COV-2 patients (51). a systematic review and meta-analysis of randomized controlled trials on the effect of Omega-3 fatty acids supplementation in patients with SARS-COV-2 have reported that Omega-3 supplementations have been associated with alleviating the inflammatory response by the CRP level reduction (52).

**Figure 2:**
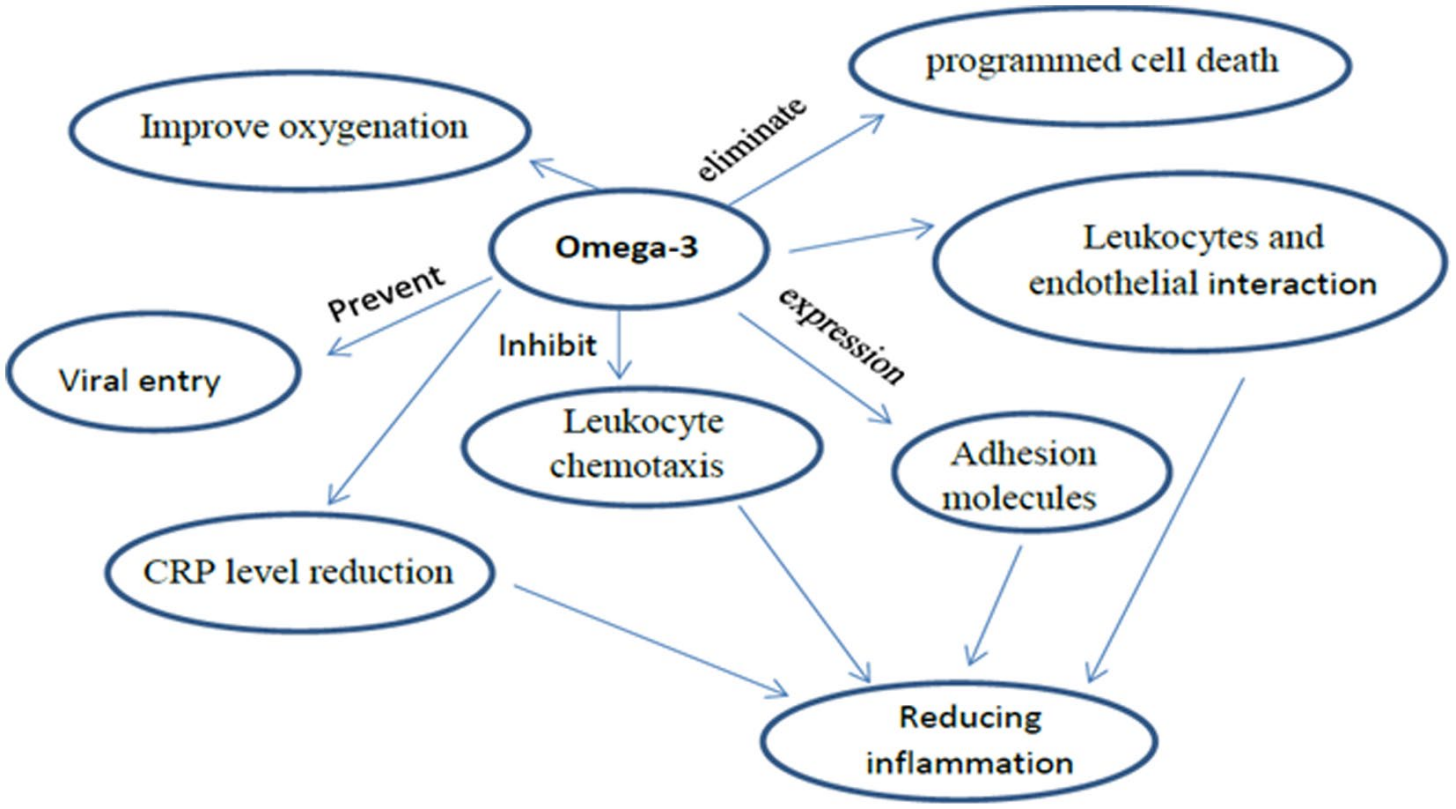
The immunomodulatory role of lipid in SARS-COV-2.

A study found that omega-3 fatty acids from marine food were correlated with lower SARS-COV-2 mortality rates. Omega-3 fatty acids could reduce SARS-COV-2 medical complications by limiting the entry of the virus into human cells *via* a mechanism that involves fatty acids binding to viral spike proteins (53). Hathaway et al. proposed that omega-3 fatty acids supplementation might inhibit entry of the virus through changes to the lipid composition in a cell’s bilipid membrane. Moreover, the omega-3 fatty acids role is critical in mediating inflammatory processes and producing a modulation effect on innate and acquired immune responses (54) (Figure 2).

Caution has been warranted with the use of omega-3 fatty acids in SARS-COV-2 patients. This is due to evidence of a counter-intuitive increase in oxidative stress and inflammation because of a higher susceptibility to damage to cell membranes (55). However, the correlation between omega-3 fatty acids and recovery in SARS-COV-2 patients is still controversial.

## Carbohydrates

**Figure 3:**
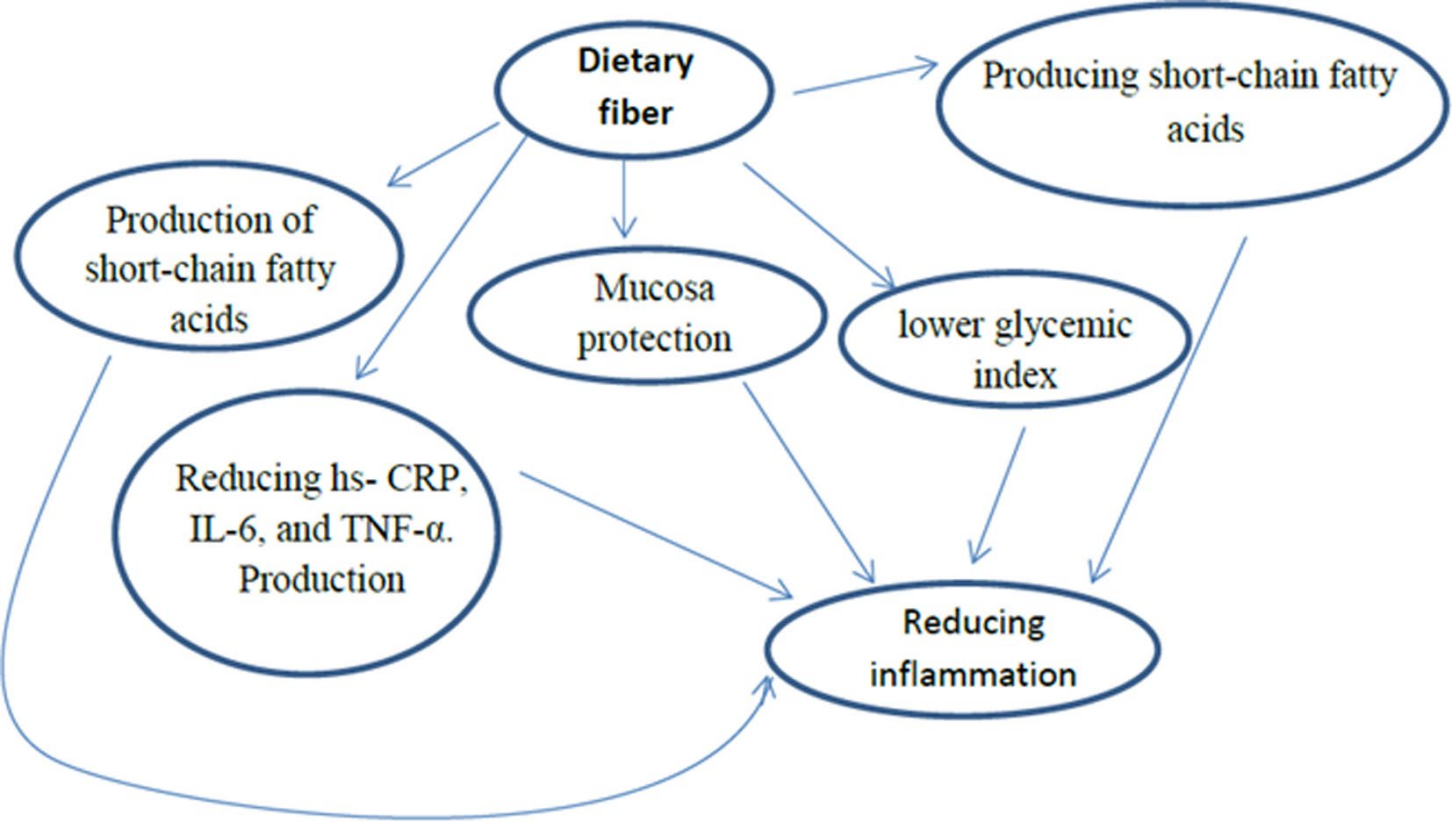
The immunomodulatory role of carbohydrates in SARS-COV-2.

Carbohydrates are mostly found in plants and used to support parts (fiber) of the plant, for plant growth, while the remaining part is for food storage (i.e., starch or sugar). Rich food sources of carbohydrates include bread, grains, vegetables, fruits, and legumes (56, 57). Carbohydrates intake should comprise 45 to 65% of total daily calories (58). Furthermore, the recommended daily fiber intake for males and females is 25 g and 38 g, respectively. A recent review study reported that the consumption of dietary fiber has anti-inflammatory effects by producing short-chain fatty acids, mucosa protection, and lower glycemic index (36). Diets with a high glycemic index produce inflammatory cytokines, TNF-α, IL-6, and C-reactive protein. Dietary fibers reduce inflammation, hs-CRP, IL-6, and TNF-α. Production of short-chain fatty acids occurs through the fermentation of carbohydrates in the gut and causes anti-inflammatory effects with up-regulated IL-10 production in monocytes (36, 57) (Figure 3). The effect of dietary fiber and prebiotic oligosaccharides is greater and typically promotes the growth of lactobacilli and bifidobacteria which benefits immunity (57). There is no evidence suggesting that carbohydrates influence SARS-COV-2 prevention and improvement (59).

## Probiotics

The human body hosts many bacteria and organisms that colonize the skin, mouth, and gut. When there is a community of organisms within a particular location is referred to as the microbiota. The colon is the site of the most significant number and variety of bacterial species (60, 61). The usual diet strongly affects the intestinal microbiota. Probiotics including lactobacillus and bifidobacterium are live microorganisms with long-term effects as part of a typical diet through fermented foods. These foods include traditionally cultured dairy products, some fermented products (dairy and non-dairy), or supplements for their beneficial effects on gut microorganisms (62). Furthermore, both aging and health significantly affect the microbiota composition. An abnormal intestine microbiota, called dysbiosis, is commonly seen in obesity and adults with various human diseases (63). Probiotics have been investigated to prevent and treat various diseases (64). It has been suggested that probiotics are beneficial for human health through their effects on strengthening the immune system (40).

### The immunomodulatory role of probiotics in SARS-COV-2

Beneficial effects from probiotics are likely the result of immune regulation, and controlling proinflammatory and anti-inflammatory cytokines (65). Studies have reported that microbiota improves resistance to viruses or pathogenic attacks of respiratory mucosa (66, 67). The results of the network and meta-analysis study showed that probiotics have therapeutic effects through many procedures such as: limiting virus entry *via* ACE2 receptor, mitigating the adverse effects of dysregulated RAS system, improving the systemic immune response, mediating Toll-like receptors (TLRs) innate immune response, improving cardiovascular complications, and reducing nitric oxide (NO) production, hypertension, and oxidative stress (68). Furthermore, another meta-analysis reported a positive correlation between probiotics intake and a reduction in COVID symptoms, especially cough, headaches, and diarrhea (69). Some specific mechanisms that explain the health benefits of probiotics include the activation of the immune reaction by interleukins and natural killer cells. Additionally, probiotics with macrophages lead to the formation of IL-12 which activates the generation of interferon-γ, considered a principal antiviral cytokine (70).

Probiotics enhance mucosal protection *via* increasing IgA production and the differentiation of CD8^+^ T cells into cytotoxic T-cells as well as CD4^+^ T cells into Th1 and Th2 cells (71). Different trials have demonstrated that probiotics exert positive effects on the gut and lungs through increases of regulatory T cells, improving anti-viral defense, and reducing pro-inflammatory cytokines during systemic and respiratory infections. Immunomodulatory benefits are crucial for SARS-CoV-2 patients or people at risk of contracting SARS-CoV-2 (72). This effect is likely the result of the gut-lung axis, which can form immune responses and disrupt respiratory diseases (73). The probiotics also affect the junction’s integrity and maintenance of the respiratory and gastrointestinal tract epithelium enterocytes, hence limiting the risk of SARS-CoV-2 entry (74). Furthermore, several probiotics especially lactic acid bacteria (LAB) produce peptides with an ACE inhibitory effect, which binds to the site of SARS-CoV-2.

A cross-sectional study on COVID-19 patients, influenza patients, and healthy controls (HC) examining gut microbiota reported that COVID-19 patients had lower gut microbiota diversity compared to HC, which was related to respiratory viral infectious diseases. Moreover, disease-specific shifts in microbiota composition were found between COVID-19 patients and HC (75). The studies on SARS-CoV-2 found significantly decreased gut bacterial diversity compared to HC, while it was increased in numerous bacterial species, such as *Collinsella aerofaciens*, *Morganella morganii*, and *Streptococcus infantis* (76, 77).

Several randomized controlled trials are underway investigating the effect of probiotic strains on SARS-CoV-2 severity, which may directly affect patients (78, 79). A study comparing probiotic-enriched formulas with placebo formula in adult symptomatic Covid19 outpatients reported that the probiotic-enriched formulas reduced symptom duration, viral load, and lung infiltrates while increasing SARS-CoV2-specific IgM and IgG in Covid19 outpatients (80). In a randomized trial, adults with symptomatic SARS-CoV-2 outpatients’ consumed probiotic formula (strains Lactiplantibacillus Plantarum KABP022, KABP023, and KAPB033, plus strain *Pediococcus acidilactici* KABP021, totaling 2 × 10^9^ colony-forming units (CFU)) or placebo for 30 days. The results showed a reduction in symptom duration, viral load, and lung infiltrate while increasing SARS-CoV-2 specific IgM and IgG (81).

**Figure 4:**
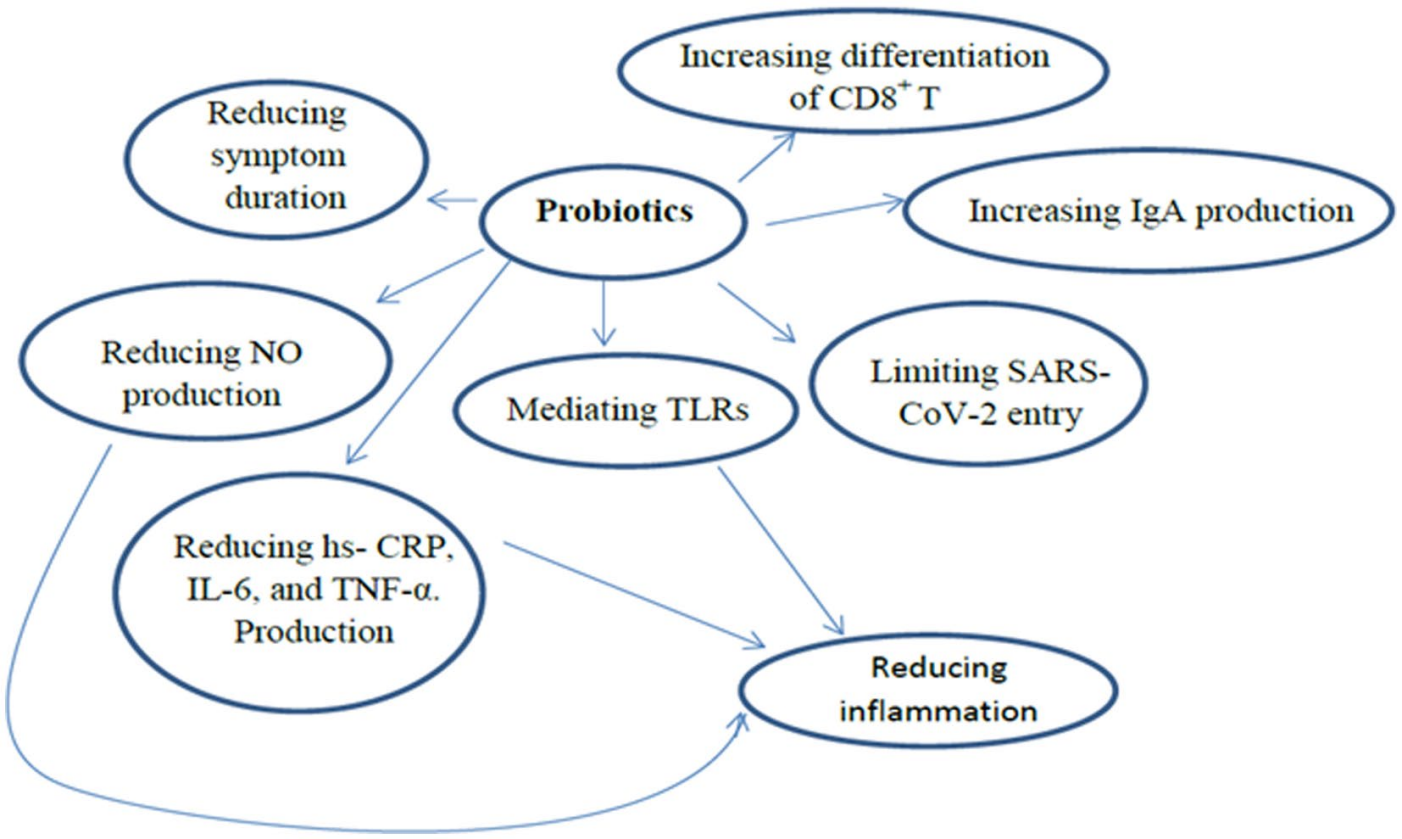
The immunomodulatory role of probiotics in SARS-COV-2.

A randomized controlled clinical trial was conducted with eighty SARS-CoV-2 patients in stages III. The control group received a hospital diet and medical treatment. The intervention group received a hospital diet, medical treatment, and the NSS (vitamins, minerals, fiber, omega-3, amino acids, B-complex, and probiotics). In the intervention group, a significant increase in survival and a decrease in mortality were observed compared to the control group (82) (Figure 4).

The authors of this study assumed that this probiotic might primarily help the gut-lung axis (GLA) by interfering with the host’s immune system. However, with the lack of evidence that probiotics affect fecal microbiota, it seems that this hypothesis cannot be confirmed. More studies are needed to confirm this hypothesis.

## Conclusion

The adequacy of globally considered macronutrients and microbiota is always fundamental for health in general and the ability of the immune system to respond adequately, not only to SARS-CoV-2, but to any other aggression by microorganisms. This article highlights the influential role of macronutrients and probiotics against SARS-CoV-2. Boosted immunity properties of macronutrients have opened a new window for their potential use for SARS-CoV-2 treatment and prognosis. Immunomodulatory agents, including macronutrients and probiotics, are among the current therapies applied in clinical settings for SARS-CoV-2. There is evidence that a diet high in protein and amino acids facilitates antibody production (83). Furthermore, a healthy balanced diet enriched in proteins, dietary fiber, and omega-3 fatty acids is highly recommended during the pandemic. Malnutrition, such as macronutrient deficiencies, is common in vulnerable groups, especially in elderly patients with multiple comorbidities and also in those groups of subjects who voluntarily submit to specific nutritional indications (for example vegan diets, ketogenic diets, vegetarian diets, food exclusion diets, etc…), or in patients affected by particular pathologies (anorexia, celiac disease, bulimia, etc…). As a result of the comorbidities and associated drugs, this may lead to tissues depleted of valuable nutrients, increase urinary excretion, or reduce nutrient absorption (37). These conditions conflict with an upcoming cytokine storm. Currently, no definitive recommendations for macronutrient and micronutrient supplementation in patients with SARS-CoV-2 exist. However, it is advised that SARS-CoV-2 patients be monitored to assess risk factors for nutrient depletion, i.e., inadequate intake and nutrient-depleting drugs. Education would also be imperative concerning depletion symptoms for laboratory monitoring of nutrients.

With numerous research groups currently involved in developing vaccines, and novel therapeutics against SARS-CoV-2, a healthy diet, balanced macronutrient status, and probiotics that reduce inflammation and oxidative stress may be an important strategy for managing SARS-CoV-2. This review highlights the role of macronutrients and probiotics in reducing inflammation, cytokine storm, and strengthening the immune system in SARS-CoV-2 patients (Supplementary Table S1).

## Author contributions

MZ, SD, AH, and AK: writing original draft. FP, AM, MZ, and SD: writing—review. NA, and DH: review and editing. All authors contributed to the article and approved the submitted version.

## Conflict of interest

The authors declare that the research was conducted in the absence of any commercial or financial relationships that could be construed as a potential conflict of interest.

## Publisher’s note

All claims expressed in this article are solely those of the authors and do not necessarily represent those of their affiliated organizations, or those of the publisher, the editors and the reviewers. Any product that may be evaluated in this article, or claim that may be made by its manufacturer, is not guaranteed or endorsed by the publisher.
