# Supplementary material for: Potential of macronutrients and probiotics to boost immunity in patients with SARS-COV-2: a narrative review
Source: Front Nutr. 2023 May 15;10:1161894. doi: 10.3389/fnut.2023.1161894 (PMC10259402; doi:10.3389/fnut.2023.1161894)
Supplement: Supplementary file 1 [file Table_1.doc]

Table 1. Nutritional significance of macronutrients

| **Macronutrients** | **Authors-published year** | **Study population** | **Study design** | **outcome** |
| --- | --- | --- | --- | --- |
| **Proteins** | Alencar etal.  2022 | 112 | retrospective | There was an association between inadequacies in protein and energy supply with mortality. |
|  | Luigi etal.  2021 | 121 | retrospective | There was a link among reduction in symptoms, age, and Lf treatment. |
|  | Ketabforoush et al.  2021 | 126 | prospective cohort study | higher amounts of protein and energy intakes in the early acute phase were significantly associated with better survival and lower risk of in-hospital mortality. |
| **Lipids** | Doaei etal.  2021 | 128 | double-blind, randomized clinical trial | Omega-3 supplementation improved the respiratory and renal function. |
|  | Sedighiyan et al.  2021 | 30 | single-blind randomised controlled trial | Omega-3 may be an appropriate adjunct therapy for management the inflammatory response |
| **Carbohydrates** | Iddir et al.  2020 |  | Review | high fiber content diet  positively impacts immune function |
| **Probiotics** | Gu etal.  2020 [76] | 84 | cross-sectional | COVID-19 patients compared with healthy controls had significantly reduced bacterial diversity, a significantly higher relative abundance of opportunistic pathogens and a lower relative abundance of beneficial symbionts. |
|  | Gutiérrez-Castrellón etal.  2022[81] | 300 | single-center, quadruple-blinded, randomized trial | Probiotic supplementation decreased viral load, lung infiltrates and duration of both digestive and non-digestive symptoms, compared to placebo. Furthermore, specific IgM and IgG against SARS-CoV2 rose. |
